# Enhancing immunomodulation on innate immunity by shape transition among RNA triangle, square and pentagon nanovehicles

**DOI:** 10.1093/nar/gku516

**Published:** 2014-08-04

**Authors:** Emil F. Khisamutdinov, Hui Li, Daniel L. Jasinski, Jiao Chen, Jian Fu, Peixuan Guo

**Affiliations:** 1 Department of Pharmaceutical Sciences, College of Pharmacy, Markey Cancer Center, Nanobiotechnology Center, University of Kentucky, Lexington, KY 40536, USA; 2 Center for Research on Environmental Disease, Graduate Center for Toxicology, College of Medicine, University of Kentucky, Lexington, KY 40536, USA

## Abstract

Modulation of immune response is important in cancer immunotherapy, vaccine adjuvant development and inflammatory or immune disease therapy. Here we report the development of new immunomodulators *via* control of shape transition among RNA triangle, square and pentagon. Changing one RNA strand in polygons automatically induced the stretching of the interior angle from 60° to 90° or 108°, resulting in self-assembly of elegant RNA triangles, squares and pentagons. When immunological adjuvants were incorporated, their immunomodulation effect for cytokine TNF-α and IL-6 induction was greatly enhanced *in vitro* and in animals up to 100-fold, while RNA polygon controls induced unnoticeable effect. The RNA nanoparticles were delivered to macrophages specifically. The degree of immunostimulation greatly depended on the size, shape and number of the payload per nanoparticles. Stronger immune response was observed when the number of adjuvants per polygon was increased, demonstrating the advantage of shape transition from triangle to pentagon.

## INTRODUCTION

One area of biomimetic nanotechnology involves the construction of nano-scale, supramolecular architectures utilizing modular units of functional nucleic acids. The aim is to design nanostructures that undergo self-assembly in a controllable fashion. Ribonucleic acid (RNA) was discovered as an attractive material to build nanoparticles *via* nanotechnology (1), offering a variety of structural modules and motifs that can be manipulated into 1D, 2D and 3D architectures (for review see (2)). In the past decade, a variety of geometric RNA nanoparticles and nano-scaffolds have been obtained *via* the approaches of hand-in-hand (1,3–5), foot-to-foot (6–9), branch extension (10–14), loop–receptor contact (15–17), ‘sticky’ or ‘dangling’ ends (6,18,19) and synthetic RNA–protein complex interactions (20). These motifs are available in databases and can be used to build artificial nanostructures by manipulating their interchangeable units (21). Recently, RNA rolling cycle transcription has been utilized to generate RNA sponges (22,23). In RNA tectonics approach, structural motifs like double helices, loops and junctions can be isolated from large and complex RNA molecules appearing in structural databases and used to build artificial nanostructures by manipulating their interchangeable units (24,25). As such, previously reported designs of RNA nanoparticles, e.g. tecto-square (26), square-shaped nano-scaffolds (27,28), RNA nano-rings (1,5,7,9) or pRNA dimmers, tetramers and hexamers (1,7,9,29,30), as well as RNA nano-cubes (19), RNA polyhedron (14), RNA bundles (6,31) and filaments (15,16) utilize fundamental principles of RNA structure and folding (32–36). Overall stability of conventional constructs though, mainly relies on the stability of canonical and non-canonical base pair (bp) forming by loop–loop, receptor–loop, or ‘sticky-ends’ with a number of pairing nucleotides usually not exceeding six. A new approach is needed to increase overall stability of RNA nanoparticles, one that uses naturally-selected stable RNA building blocks for structure building, and the example is the 3WJ motif from pRNA of bacteriophage phi29 DNA packaging motor. In addition to discovering that the pRNA-3WJ shows exceptional stability under physiological conditions and in the presence of strong denaturing agent (10,11), recent studies also suggest that the thermodynamic stability of the 3WJ is entropy driven (37).

**Figure 1.**
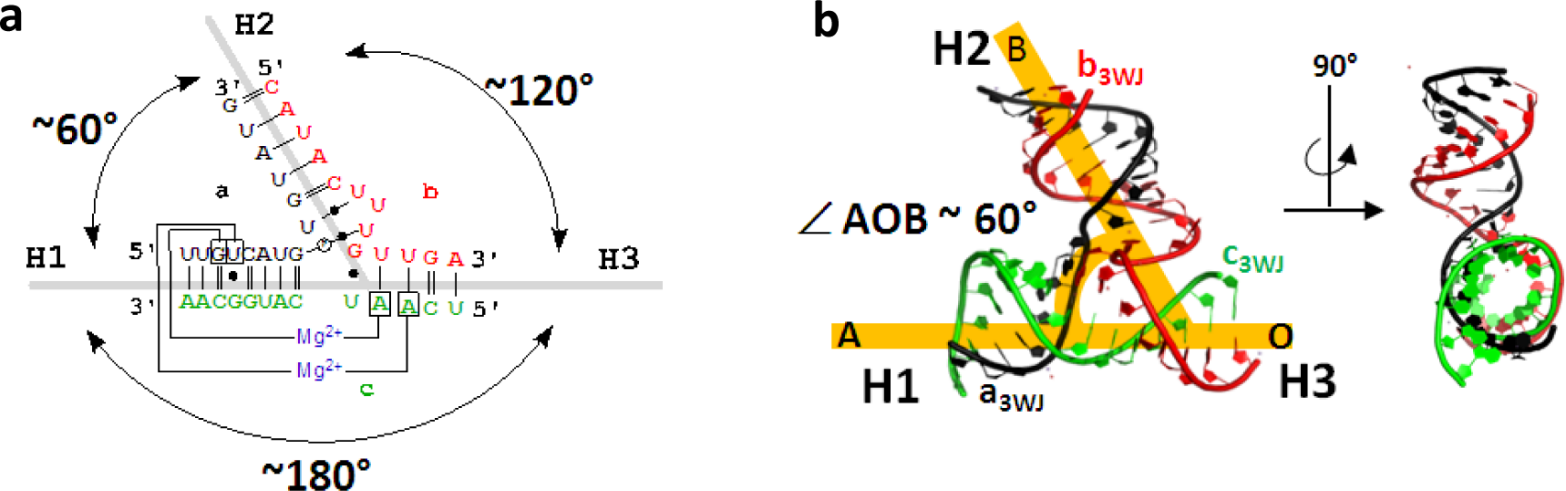
Structural features of the pRNA 3WJ motif. (**A**) Secondary structure of 3WJ motif with base pairs annotated using Leontis–Westhof nomenclature (25). (**B**) Tertiary structure of 3WJ motif with indication of ∠AOB ∼ 60° angle formed between H1 and H2. Angle corresponds to inner angles of polygons.

In this report, we introduce a conceptual approach to the rational design of stable RNA architectures by stretching the 60° AOB angle (∠) (Figure 1) of the thermodynamically stable pRNA 3WJ motif. We demonstrate that it can be stretched to wide conformations resulting in different 2D polygons: triangle (∠AOB = 60°) (18), square (∠AOB = 90°) and pentagon (∠AOB = 108°). Intermolecular interactions such as kissing loops, receptor loop, or ‘sticky-ends’ were avoided by introducing linkages through base pairing between corners of the polygons using RNA double helices. Therefore, this system is advantageous with an increased thermo-stability in the overall construct.

We further demonstrate that the RNA polygons have the potential to serve as a new generation of delivery systems for immunomodulators. Synthetic unmethylated cytosine-phosphate-guanine oligodeoxynucleotides (CpG ODN) are immunostimulatory DNA molecules that mimic the immunostimulatory activity of bacterial DNA (38,39). CpG DNA motifs strongly activate the mammalian innate immune system by interacting with various immune cells *via* endosomal Toll-like receptor 9 (TLR9) (40,41). Upon the stimulation by CpG DNA, immune cells could secret a variety of proinflammatory and antiviral cytokines including tumor necrosis factor-α (TNF-α), interleukin-6 (IL-6), IL-12 and interferon (IFN), which leads to potent immune response. The therapeutic potential of CpG DNA has also been extensively explored in both basic research and human clinical trials, including the development of new vaccine adjuvants, anticancer agents, immunoprotective agents and anti-allergic agents (42–45). Recently, several groups have reported the utilization of DNA nanostructures such as DNA tetrahedrons (46), DNA polypod-like structures (47), DNA origami structures (48), Y-shaped DNA (49) and DNA dendrimers (50) to deliver immunostimulatory CpG DNA. In this work, to the best of our knowledge, we report the first use of RNA nanostructures to deliver CpG DNA *in vitro* and *in vivo*. The immunostimulatory efficacy of RNA polygons was evaluated by measuring the release of cytokines. We found that the induction of cytokines is highly dependent on the number of CpG per polygon. With increasing number of CpG per polygon, stronger immune response was observed, demonstrating the advantage of the transition from a triangle to a pentagon that can carry five CpGs.

## MATERIALS AND METHODS

### RNA nanoparticles design, synthesis and self-assembly

The 3WJ crystal structure of the pRNA molecule (PDB ID: 4KZ2) was primarily used for designing polygon models using Swiss PDB viewer, as previously described (18). RNA strands for corresponding triangle, square and pentagon, were synthesized by *in vitro* T7 transcription using polymerase chain reaction (PCR) generated DNA templates. RNAs were purified by denaturing polyacrylamide gel electrophoresis (PAGE) and were either Cy5 whole body RNA labeled (Mirus Bio LLC) or 5′-end [γ-^32^P] ATP (PerkinElmer) labeled, as previously described (8).

RNA polygons were assembled in one pot by mixing equimolar concentrations (1 μM) of four RNA strands for the triangle, five RNA strands for the square and six RNA strands for the pentagon in 1× TMS buffer (50 mM TRIS pH 8.0, 100 mM NaCl and 10 mM MgCl_2_). Samples were annealed for 1 h in a thermocycler with controlled, slow cooling (1°C/min) from 80 to 4°C. All RNA polygons harboring CpG ODNs were assembled from their corresponding 2′F-U/C modified strands in one pot procedure.

### Native PAGE, temperature gradient gel electrophoresis (TGGE) and boiling resistance assays

RNA assemblies were evaluated on 7% (29:1) native polyacrylamide gels in the presence of 0.5 × TMS buffer. Gels were run at constant 90 V, +4 °C. Gels were imaged with Typhoon FLA 7000 (GE Healthcare) to visualize RNA strands. TGGE analysis was performed on 7% native PAGE in a buffer containing 50 mM TRIS pH 8.0, 100 mM NaCl and 0.2 mM MgCl_2_, as previously described (14,26,28). A gradient temperature of 30–70 °C was applied perpendicular to electrical current and the experiment was run for 1 h at 20 W. A total RNA concentration of 100 nM was used in TGGE analysis. Apparent *T*_M_ values corresponded to the temperature at which half of the polygons fractions were dissociated and apparent *K*_D_ values for multiple RNA strands were calculated, as described previously (7).

Boiling resistance assay was performed in 10 μl containing 1 μM preassembled polygons in TMS buffer or in the presence of 8 M urea. Samples were incubated at 100 °C for several minutes, then snap cooled on dry ice to prevent refolding following evaluation on 7% native PAGE at 4 °C. Individual experiments were repeated several times to reduce error.

Quantification analysis was performed using ImageJ (52). Equal-sized boxes were drawn around the lanes corresponding to the triangle, square, or pentagon complexes and corresponding quantified values for each type of polygon were divided by the sum of the values presented in the corresponding lane.

### Cell cultures

Mouse macrophage-like RAW 264.7 cells were grown in Dulbecco's Modified Eagle's Medium supplemented with 10% fetal bovine serum, 100 units/ml penicillin and 100 mg/ml streptomycin at 37°C in humidified air containing 5% CO_2_. Cells were then seeded on 24-well plates or 96-well plates at a density of 5 × 10^5^ cells/ml and cultured overnight before use.

### Cytokine secretion from RAW264.7 Cells

RAW 264.7 cells were plated into 24-well plates with the density of 2.5 × 10^5^ cells per well and cultured overnight. Then, RNA nanoparticles harboring different numbers of CpG ODNs were diluted in Opti-MEM medium (Life Technologies Corporation, Carlsbad, CA, USA) and added to the cells. The cells were continually cultured for 8 h at 37 °C in humidified air containing 5% CO_2_, and the cell culture supernatant were collected and stored at −80 °C until use. The concentration of TNF-α and IL-6 in the supernatant were determined by enzyme-linked immunosorbent assay (ELISA) using Mouse ELISA MAX™ Deluxe sets (BioLegend, Inc., San Diego, CA), following protocols provided by the manufacturer.

### Cytokine secretion from mice

Male CD-1 mice (4–5 weeks old) were purchased from Charles River Laboratories. All animal procedures were approved by the Institutional Animal Care and Use Committee at University of Kentucky and were performed in accordance with guidelines issued by the National Institutes of Health for the care of laboratory animals. For *in vivo* immunostimulation, RNA triangular nanoparticles harboring CpG ODN, RNA triangular nanoparticles, or CpG ODN were dissolved in phosphate buffered saline (PBS) and administrated to the mice via tail vein injection at 2 mg/kg (CpG ODN per body weight). The same volume of PBS was injected into a mouse as a control. Blood samples were collected 3 h post-injection by cardiac puncture. Serum was prepared by centrifugation at 12 800 g for 10 min. Serum TNF-α and IL-6 levels were determined by enzyme-linked immunosorbent assay (ELISA) using Mouse ELISA MAX™ Deluxe sets (BioLegend, Inc., San Diego, CA, USA), following protocols provided by the manufacturer.

### Confocal microscopy imaging

RAW 264.7 cells were seeded on glass coverslips in 24-well plates and cultured at 37 °C in humidified air containing 5% CO_2_ overnight. The culture medium was removed and the cells were washed with Opti-MEM medium twice to remove dead cells. RNA nanoparticles harboring Cy3-labeled CpG DNA or Cy3-labled CpG DNA only were diluted in Opti-MEM medium and added to the cells. After 4 h incubation at 37 °C in humidified air containing 5% CO_2_, the cells were washed twice with PBS and fixed with 4% formaldehyde. ProLong® Gold Antifade Reagent with DAPI (Life Technologies Corporation, Carlsbad, CA) was used to stain the cell nucleus and mount the samples. Alexa Fluor^®^ 488 phalloidin (Life Technologies Corporation, Carlsbad, CA, USA) was used to stain actin. The images were obtained on a Olympus FV1000 confocal microscope (Olympus Corporation, Tokyo, Japan).

## RESULTS

### RNA polygons: triangle, square and pentagon fabrication and self-assembly

**Figure 2.**
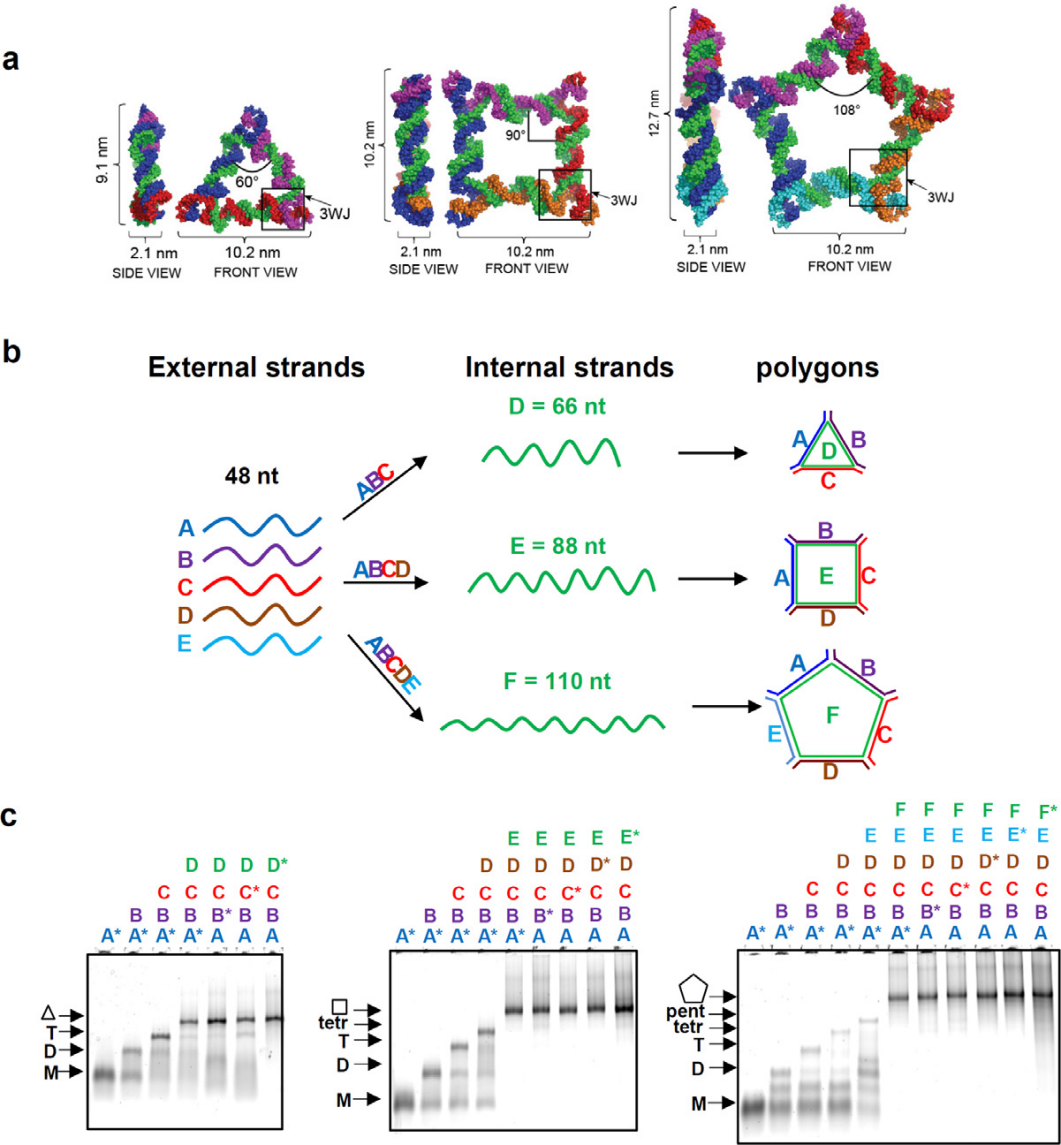
Design of Polygons and assembly properties. (**A**) 3D modeled structures of polygons with 3WJ motif located at vertices, inner angle corresponds to ∠AOB. (**B**) The increasing length of the internal strand (green) stretches the 3WJ ∠AOB at which the nanoparticles assemble, along with increasing number of external ‘short’ strands. (**C**) Assembly properties of polygons evaluated on 7% native PAGEs. Asterisks ‘*’ indicate the Cy5 labeled strands utilized on each type of polygon assembly.

The structural features of the recently discovered ultrastable pRNA 3WJ module from the bacteriophage Phi29 DNA packaging motor were utilized for *in silico* design of the RNA triangle, square and pentagon 2D polygons. During the computer modeling we used the particular angle of the 3WJ formed by H1 and H2 as an inner angle of the polygons as we hypothesized that the angle could be stretched to a more open conformation. Throughout this report the intra-helical angle between H1 and H2 is denoted as ∠AOB, as shown in figure 1. Each RNA model contained a pRNA 3WJ motif at each vertex, and the inner angles correspond to ∠AOB. The resulting 3D models exhibited flat conformations, as expected from the plane geometry of the 3WJ motif (51) (Figure 2A).

Each polygon was composed of a different number of RNA strands classified as short strands (external) and long strands (internal) (Figure 2B). By increasing the number of external strands and the propagation of the central or internal strand, the tension on the inter-helical ∠AOB increased to 60°, 90° and 108°, allowing for 2D formation of corresponding triangle, square and pentagon shapes. The measured widths, from one corner to another, were 10.2 nm, while the heights differed as follows: triangle = 9.1 nm, square = 10.2 nm and pentagon = 12.7 nm. Following the transcription of individual RNA strands, self-assembly properties of the triangle, square and pentagon designs were evaluated on 7% native polyacrylamide gel electrophoresis (PAGE) (Figure 2C). All polygon formations were obtained by one-step self-assembly (7,10,11,19). Each RNA component of corresponding nanoparticles were whole chain labeled with Cy5 to evaluate participation of all RNA strands in their corresponding assemblies. Yield of correctly assembled polygons was estimated to be >90% based on native PAGE gel evaluations. Equilibrium constants of dissociation were obtained from apparent *K*_D_ gels, and *K*_D_ values were determined to be 18.8, 20.3 and 22.5 nM for triangles, squares and pentagons, respectively (Supplementary Figures S1 and S2).

These results demonstrate that each RNA nanoparticle assembles into the desired nanostructure, and indicated by a stretching of the 60°∠AOB to wider conformations. The assembly of RNA strands into specific-shaped nanoparticles based on the 60°∠AOB of the pRNA 3WJ motif was controlled by modulating the number of short external stands and the length of the long internal strand.

### Structural characterization of polygons by atomic force microscopy (AFM) and dynamic light scattering (DLS)

**Figure 3.**
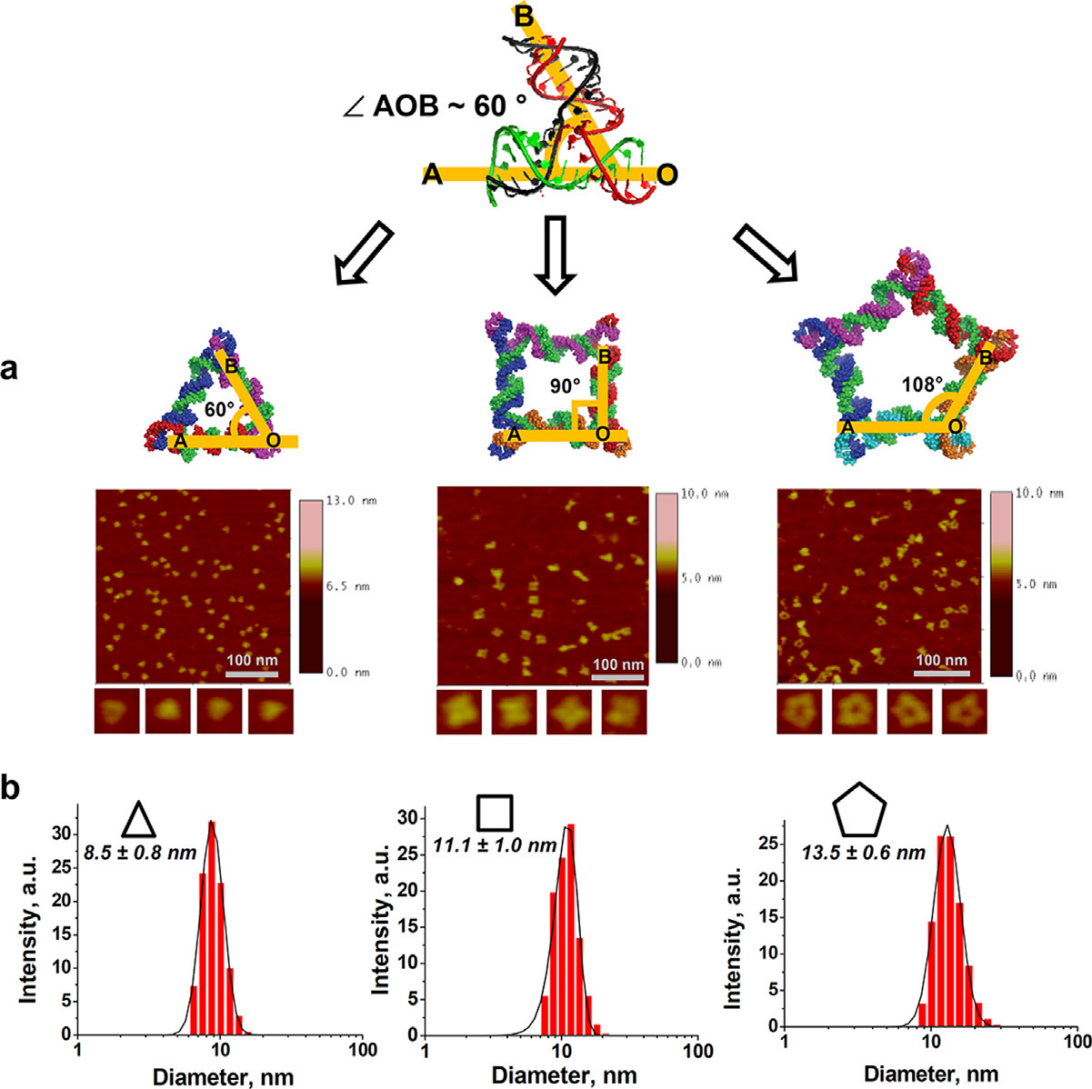
Structural characterization of polygons. (**A**) AFM images of triangular, square and pentagonal nanoparticles derived from the pRNA 3WJ motif. (**B**) Polygons size distribution histogram obtained *via* dynamic light scattering.

To further evaluate the size and shape of the resulting RNA assemblies, structural characterization of each polygon was conducted by AFM. AFM images of the pRNA 3WJ based polygons revealed that the shapes of resulting polygons were similar to their predicted, theoretical 3D models (Figure 3A). The estimated average dimensions were found to be 13 ± 1.1, 14 ± 1.8 and 17 ± 1.6 nm for triangles, squares and pentagons, respectively. These values do not reflect the true sizes of the RNA polygons due to the AFM tip convolution, but rather demonstrate that the average size of the nanoparticles increases from triangle to pentagon. In addition, the central cavity of each RNA shape is visible, and the size of the cavity gradually increases with the number of polygon vertices. The measured heights for all nanoparticles was found to be ∼2 nm, in agreement with previously reported heights of nucleic acid double helices (26,53).

Quantification analysis was performed to compare apparent yields between polygons observed on AFM mica surface. Equal concentrations (1 nM) of the polygons were deposited on a mica surface and correctly folded polygons were manually counted in a 0.5 μm^2^ area, resulting in 48 triangular particles, 33 square particles and 17 pentagon nanoparticles (Supplementary Figure S3). The estimated number of triangular nanoparticles adsorbed on the mica was ∼1.5 times more than that of the square and three times more than that of the pentagon. However, native PAGE revealed that the yields among the three polygons were almost equal. The difference in adsorption amounts between polygons on the mica surface was presumably due to variation in their sizes and 3D conformations, resulting in different dynamic and physical properties.

DLS was performed to determine the apparent hydrodynamic diameters for each of the polygons. The diameters were found to be 8.5, 11.1 and 13.5 nm for triangles, squares and pentagons, respectively (Figure 3B). The increase in number of 3WJ cores corresponds with the larger observed diameter. The measured diameters agreed with their corresponding 3D models. However, there was a discrepancy between polygon sizes determined by AFM and DLS. This could be attributed to the fundamentally different techniques, as DLS determines the average size distribution profile of nanoparticles in solution assuming that the polygons have globular shapes [refer to manual at http://www.malvern.com], while AFM imaging can produce images larger than the real diameter due to tip size used (54) and the resolution of imaging equipment. Nevertheless, the two techniques demonstrated that the relative size of the nanoparticles increased from triangle to square to pentagon.

Accordingly, native PAGE, AFM and DLS showed the formation of compact molecular 2D assemblies of triangle, square and pentagon based on the pRNA 3WJ ∠AOB. Consequently, the naturally preserved 60° ∠AOB could be stretched to reach the formation of square and pentagon. The stretching, or tension, that the angle underwent could have had a significant impact on the overall stability of the nanoparticles. Therefore, it was of great interest to evaluate and compare the polygon's stabilities.

### Stability comparison between triangle, square and pentagon

**Figure 4.**
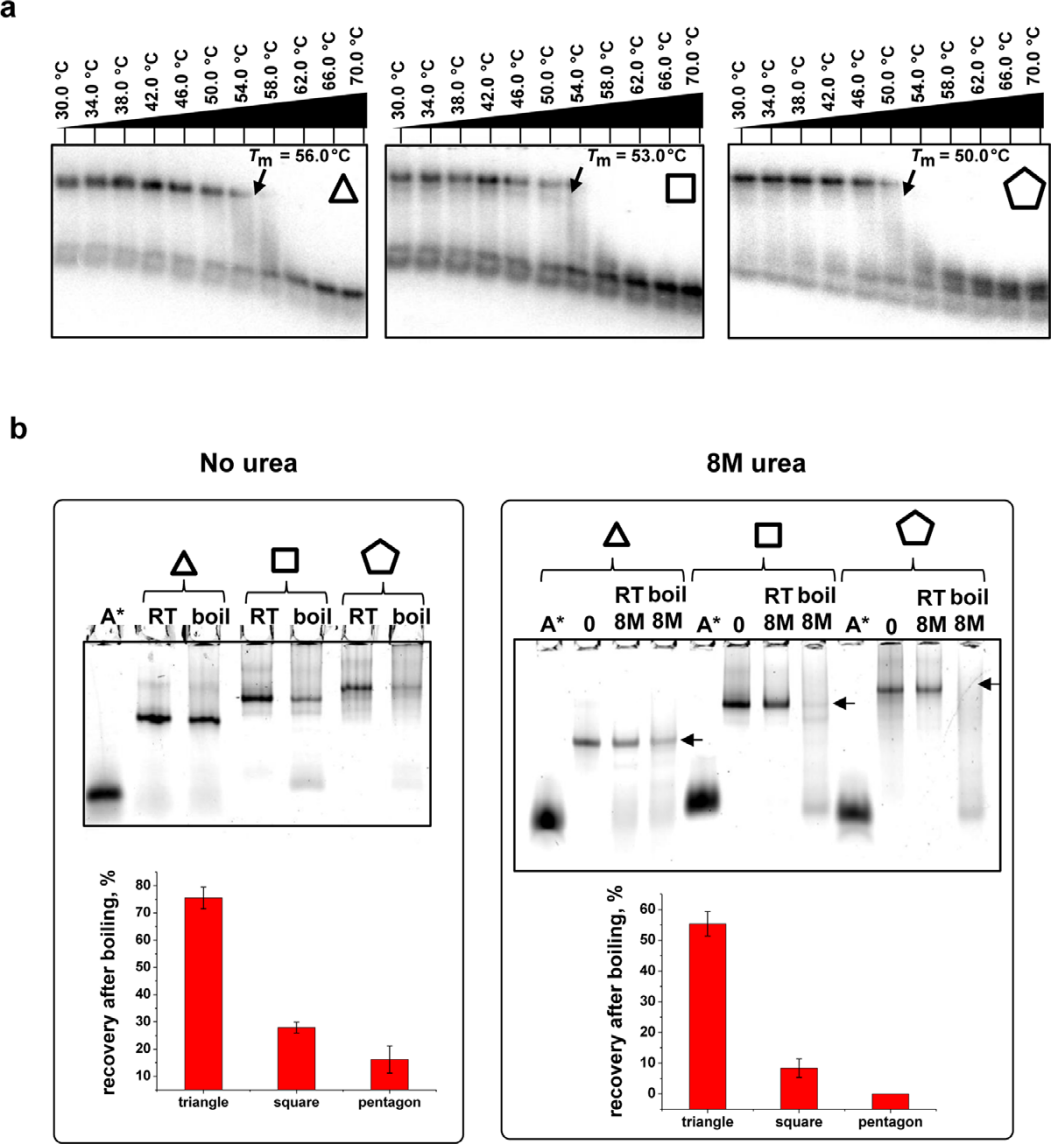
Comparison of polygon stabilities. (**A**) Melting temperatures of triangle, square and pentagon assessed by 7% perpendicular TGGE. (**B**) Boiling resistance assay in absence and presence of 8 M urea. Calculated percentage of recovery for polygons after boiling is shown below each gel with error bars calculated from several independent experiments.

The stabilities of polygons were studied using a perpendicular TGGE (Biometra GmbH). This convenient technique has garnered widespread use for measuring melting temperatures of RNA nanoparticles with multiple strands (5,7,14,26,28,55). The preassembled polygons were subjected to 7% native TGGE with a gradient temperature of 30–70°C perpendicular to electrical current. The following apparent *T*_M_ values were obtained for the polygons at 100 nM total concentration (Ct) in presence of 0.2 mM MgCl_2_: triangle *T*_M_ = 56°C, square *T*_M_ = 53°C and pentagon *T*_M_ = 50°C (Figure 4A). The triangular nanoscaffolds were more stable than squares and pentagons, although the number of RNA bp was much higher in the pentagon construct, as compared to the square and triangle. Usually, the stability of nucleic acids with the same base-pair content is directly dependent on metal ion and total nucleic acid concentrations. Since these two criteria were the same, it was assumed that the higher the number of bp in a given RNA structure the higher the stability. Therefore, the most stable shape produced should be the pentagon. However, based on TGGE data the opposite was found. This was likely due to the tension caused by the stretching of the native pRNA 3WJ 60° ∠AOB. The triangular construct angle was preserved (60°), the square and pentagon angles were stretched to the wider conformations of 90° and 108°, respectively. Previously it has been shown that any nucleotide mutations or deletions within the native core structure of the pRNA 3WJ motif would also result in the loss of its thermodynamic stability (10). Interestingly, the measured triangle and square *T*_M_ values differed by +3°C, as did the square and pentagon. Boiling resistance assay in the presence and absence of 8 M urea further confirmed that the triangle was the most stable nanoparticle (Figure 4B). The quantification of nanoparticle bands after heating to 100°C resulted in 75 ± 4% recovery of the triangular assembly, suggesting a *T*_M_ >100°C. By definition, *T*_M_ is the measured temperature when half the RNA concentration has melted, i.e. 50% recovery. The percentage of recovery for square was 28 ± 2% and for pentagon was 16 ± 5%, much lower than the value estimated for triangle recovery. The experiment with the presence of 8 M urea in boiling solution showed that the overall trend of stability remained the same, but the percentage of recovery was 55 ± 4% for triangle, 8 ± 3% for square and no pentagon fraction was detected. Overall, the nanoparticle with fewer 3WJ motifs (triangle) resulted in a higher thermostability and resistance in chemical degradation and the change in stability was in large part due to the stretching of the ∠AOB.

### The modulation effect of triangle, square and pentagon harboring immunological adjuvant

CpG oligonucleotide is an immunological adjuvant popularly used as vaccine adjuvant or immunotherapy reagent for disease control and treatment (42). To evaluate whether RNA polygons can enhance the immunomodulation effect, CpG oligonucleotide was incorporated with each RNA polygon using one-pot self-assembly (Supplementary Figures S4–S6). The toxicity assay for the resulting complexes revealed no toxicity; moreover the RNA polygon-CpG complexes induced cell proliferation during the incubation period, as compared to the cell only control (Supplementary Figure S7).

**Figure 5.**
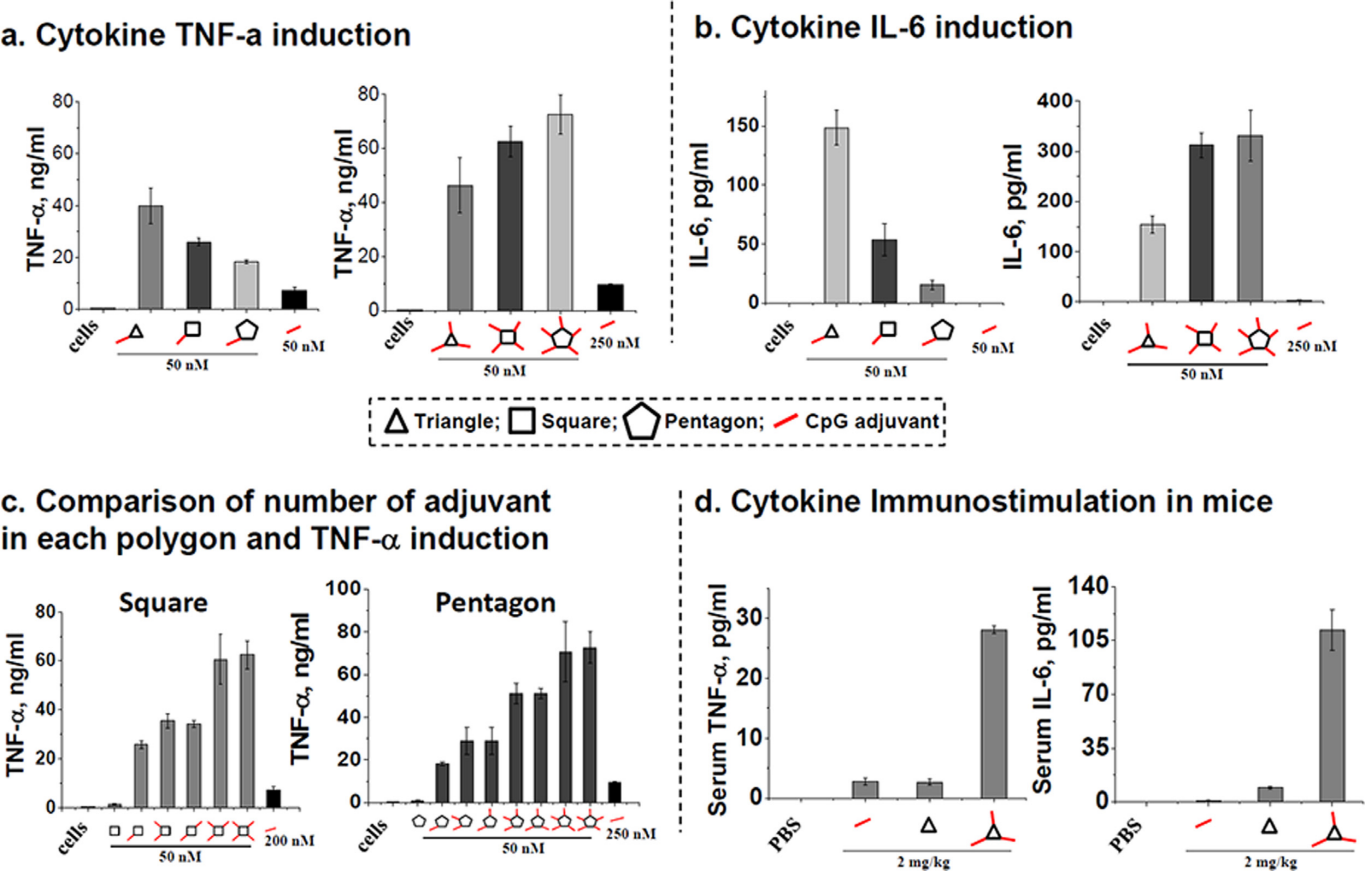
Effect of cytokine induction in macrophage-like RAW 264.7 cells and mice by RNA-CpG polygons. Induction of (**A**) TNF-α and (**B**) IL-6 cytokines by 50 nM RNA polygons-CpG. (**C**) Dependence of TNF-α induction with the number of CpGs per RNA polygon. The error bars represent standard deviation from at least three independent experiments. (**D**) Immunostimulatory activity by triangle-CpG nanoparticle in animal model. The error bars represent standard deviations of two independent measurements of the cytokine levels from serum aliquots of the tested mouse.

The extracellular immunostimulatory efficacy of RNA polygons was evaluated by measuring the release of cytokines TNF-α and IL-6 after addition to mouse macrophage-like RAW 264.7 cells (Figure 5A and B), as previously described (56,57). The triangular RNA nanoparticle coupled with only one CpG exhibited the highest level of cytokine induction for both TNF-α and IL-6 compared to square and pentagonal RNA nanoparticles. Increasing the number of CpG per nanoparticle yielded the opposite effect, as pentagonal RNA nanocarriers showed the highest level of the induction of both TNF-α and IL-6 presumably due to the increased local CpG concentration. The results suggest that the cytokine release by CpG coupled to RNA polygons with different shapes remarkably increases the immunostimulatory activity compared to CpG alone (Figure 5). RNA particles with the size of about 10 nm, such as the triangle, induced the greatest amount of TNF-α and IL-6. In addition, the induction of cytokines was highly dependent on the number of CpG per polygon. With increasing number of CpG per polygon, a stronger immune response is observed (Figure 5C), demonstrating an advantage of transiting from triangle to pentagon that can carry five CpG oligonucleotides.

### Enhancement of modulation effect in animal by immunological adjuvant incorporated into RNA triangle

To examine whether RNA nanoparticles retain their immunostimulatory activity *in vivo*, nanoparticles were administered to CD-1 mice by injection into the tail vein at 2 mg/kg (CpG oligonucleotide per body weight), following level determination of cytokine TNF-α and IL-6 levels after 3 h post-administration in collected blood serum. Figure 5D shows that free triangle nanoparticles and free CpGs did not induce any cytokine production, whereas the complex triangle-CpG resulted in elevated levels of both cytokines. The difference in immunostimulatory activity of triangle-CpG was estimated to be 10-fold compared to free CpG *in vivo*. These data are in agreement with the *in vitro* stimulation of murine RAW 264.7 cells.

### Comparison of cellular uptake by different CpG-RNA polygons

**Figure 6.**
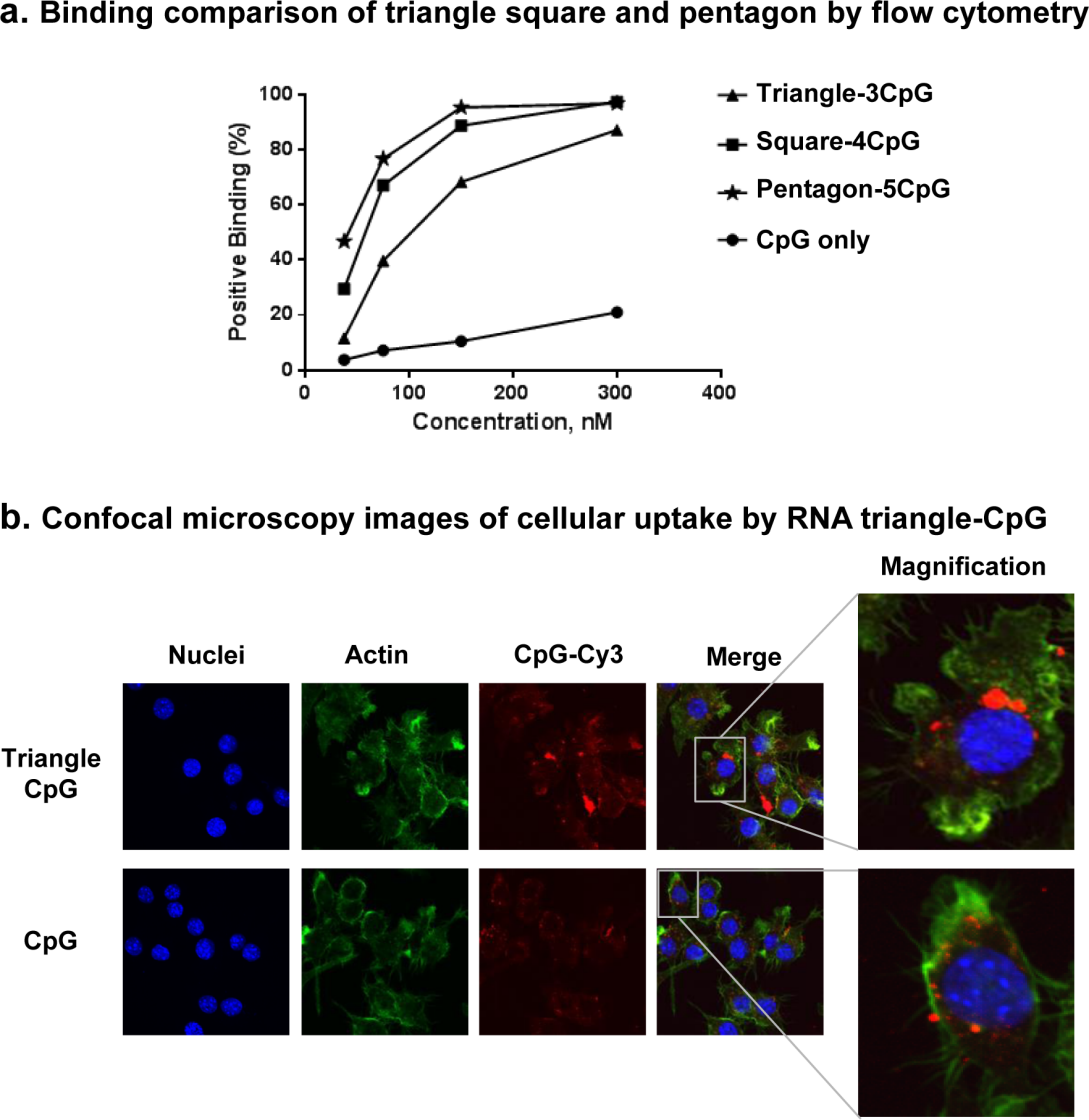
Comparison of RNA polygon-CpG complexes binding to the cells. (**A**) The plot represents the summary of the flow cytometry data showing each RNA nanoparticle-CpG adjuvants binding to the cell in a dose-dependent manner. (**B**) Confocal images showing the binding comparison of the triangle-CpG and CpG to the RAW 264.7 cells by colocalization of nucleus (blue), actin or cytoplasm (green) and Cy-3-labeled CpGs (red) signals.

Previously, it has been demonstrated that the CpG oligonucleotide can be readily recognized by TLR9 on the endosomal membrane of macrophages, resulting in cellular uptake of the CpG adjuvants (58,59). To investigate whether there is a difference between the efficiency of RNA polygons binding to the cells, we quantified the cellular uptake of polygons-CpG using flow cytometry assay (9,10). Figure 6A demonstrates the binding of different RNA polygon-CpG to the RAW264.7 cells in a dose-dependent manner. There was an increase in binding efficiency from triangle to pentagon with more CpGs (Supplementary Figure S8). Notably, all RNA polygons-CpG complexes remain intact after 16 h incubation in 10% fetal bovine serum (FBS) indicating robustness of the assembled complex in extracellular environment (Supplementary Figure S9). Overall, RNA polygon-CpG complexes exhibit significantly more binding efficiency to cells compared to CpG oligonucleotides alone.

This observation was further confirmed by confocal microscopy images (Figure 6B) revealing that RNA nanoparticle harboring CpGs are localized in the cytoplasm exclusively and that there are much higher amounts of triangular nanoparticles inside the cell compared to free CpG, suggesting that RNA nanoparticles could efficiently enhance the cellular uptake of the CpG adjuvants.

Collectively, both flow cytometry and confocal imaging demonstrated that all RNA nanoparticles with different shape and harboring CpGs have much stronger binding and cellular entry to the macrophage-like RAW 264.7 cells compared to free CpGs. In addition, cellular uptake was highly dependent on the number of CpG per polygon. With the increasing number of CpG per polygon, more efficient cell binding and entry was observed (Figure 5C and Supplementary Figure S8), demonstrating the advantage of the transition from triangle to pentagon nanoparticles that can carry five CpG.

## DISCUSSION

Artificial construction of RNA nanoparticles requires knowledge of the structural properties of RNA motifs, such as interhelical or intrahelical distances, X–Y and X–Y–Z angles, number and orientations of RNA branches in multi-way junctions, canonical and non-canonical interactions, binding sites for proteins, metal ions and small molecules (2,10,16,25,51,60). Progression in RNA structural biology allowed for the analysis of RNA 3D motifs from existing RNA structures at atomic resolution and various databases, including www.pdb.com, RNA multi-way junction (61) and Find RNA 3D motif (62). This study, based on the previously reported versatile pRNA 3WJ 3D motifs (PDB accession ID: 4KZ2) (10,51), shows that the 3WJ structure can be folded into desired conformations based on the dynamics of the 60°∠AOB, demonstrating the ability to tune the physical and structural properties of RNA polygons for a variety of technological, biological and medicinal needs. By using the 3WJ, a strategy has been reported to tune the size of a square by altering the length of each side of the polygon (63). This approach, based on the propagation of the central RNA strand used to direct the folding of corresponding short or external strands into planar triangle, square and pentagon conformations, resulted in the 60º, 90º and 108º bending of interhelical ∠AOB of the pRNA 3WJ. This is especially important for medical applications where one needs to construct different RNA nano-scaffolds based on a non-toxic and thermodynamically stable building block. The following advantages result from this technique: (i) the number and combination of therapeutic molecules can be tuned to an RNA-based nano-carrier; (ii) the nano-scaffold has a controllable size and shape and (iii) variable thermodynamic and RNase resistance properties can be applied depending on the application of the nano-scaffold.

In addition to the discovery of the unique approach for the rational design of stable RNA nanoparticles, it has been demonstrated that each RNA polygon has the potential to serve as multivalent nanocarriers for vaccine adjuvants, particularly of CpG oligodeoxynucleotides. The designed RNAs self-assemble into distinct, non-toxic homogeneous nanoparticles with high chemical, thermal and intracellular stability. We found that the size and shape of the RNA nanostructures are important factors for the induction of immunostimulatory processes *in vitro* and in animal models and that there is a correlation between the cytokine induction and the local CpG concentration effect. The highest level of secretion of pro-inflammatory cytokines TNF-α, IL-6 was obtained with the smallest nanoparticle (triangle ∼ 9 nm size) harboring one CpG compared to square (∼11 nm) and pentagon (∼13 nm). However, upon increasing the numbers of CpG per RNA nanoparticle the cytokine induction was affected more by pentagon as the number or local concentration of CpG is highest in the pentagon. This study illustrates the importance of the size and shape of RNA nanoparticles for improvement of activity of CpG based vaccines adjuvants targeting infectious diseases and cancer cells, as well as for increasing immune responses by the innate and adaptive immune systems. The RNA nanoparticles harboring CpGs are safe, effective, versatile and easy to manufacture, offering new solutions to address the unmet needs in current vaccines and adjuvants design and development. Recent findings on the thermodynamically ultra-stable (10,11) and heat-resistant (18) RNA nanoparticles have expanded the potential for application of pRNA 3WJ derived nanoparticles in the fields of biomedical, nanotechnology, or polymer industries.

## ACCESSION NUMBER

PDB ID: 4KZ2.

## SUPPLEMENTARY DATA

Supplementary Data are available at NAR Online.

SUPPLEMENTARY DATA
