# Supplementary material for: Enhancing immunomodulation on innate immunity by shape transition among RNA triangle, square and pentagon nanovehicles
Source: Nucleic Acids Res. 2014 Aug 4;42(15):9996–10004. doi: 10.1093/nar/gku516 (PMC4150753; doi:10.1093/nar/gku516)
Supplement: SUPPLEMENTARY DATA [file supp_42_15_9996__index.html]

Enhancing immunomodulation on innate immunity by shape transition among RNA triangle, square and pentagon nanovehicles — Enhancing immunomodulation on innate immunity by shape transition among RNA triangle, square and pentagon nanovehicles — SUPPLEMENTARY DATA 

# Enhancing immunomodulation on innate immunity by shape transition among RNA triangle, square and pentagon nanovehicles

## SUPPLEMENTARY DATA

**Files in this Data Supplement:**

- SUPPLEMENTARY DATA
