# Supplementary material for: Enhancing immunomodulation on innate immunity by shape transition among RNA triangle, square and pentagon nanovehicles
Source: Nucleic Acids Res. 2014 Aug 4;42(15):9996–10004. doi: 10.1093/nar/gku516 (PMC4150753; doi:10.1093/nar/gku516)
Supplement: SUPPLEMENTARY DATA [file supp_gku516_nar-00640-r-2014-File008.docx]

**SUPPLEMENTARY INFROMATION:**

Enhancing Immunomodulation on Innate Immunity by Shape Transition Among RNA Triangle, Square, and Pentagon Nanovehicles

Emil F. Khisamutdinov^1†^, Hui Li^1†^, Daniel L. Jasinski^1†^, Jiao Chen^2^, Jian Fu^2,3^ and Peixuan Guo^1^**^*^**

^1^Department of Pharmaceutical Sciences, College of Pharmacy, Markey Cancer Center, Nanobiotechnology Center, University of Kentucky, Lexington, KY 40536, USA

^2^Center for Research on Environmental Disease,  University of Kentucky, Lexington, KY 40536, USA

^3^Graduate Center for Toxicology, University of Kentucky, Lexington, KY 40536, USA

^†^Contributed equally

***Address correspondence to:**

Peixuan Guo

Email: [Peixuan.guo@uky.edu](mailto:Peixuan.guo@uky.edu)

Table of contents:

1. **Material and Methods**
2. **Supplementary figure 1**. RNA polygons dissociation constant determination at equilibrium state.
3. **Supplementary figure 2**. Sequences and secondary structures of RNA polygons.
4. **Supplementary figure 3**. AFM images of RNA polygons. AFM images of RNA polygons. **Supplementary figure 4.** Secondary structure of triangle-CpG nanoparticle and characterization.
5. **Supplementary figure 5.** Secondary structure of square-CpG nanoparticle and characterization.
6. **Supplementary figure 6**. Secondary structure of pentagon-CpG nanoparticle and characterization.
7. **Supplementary figure 7**. Effect of RNA-CpG adjuvants on RAW264.7 cell viability
8. **Supplementary figure 8**. Binding effect of RNA polygons harboring CpG adjuvants to RAW264.7 cells.
9. **Supplementary figure 9**. Serum stability assay of RNA polygons coupled with CpGs motifs

**MATERIAL AND MEHTODS**

**Atomic force microscopy imaging**

RNA polygons were imaged with MultiMode AFM NanoScope IV system (Veeco), as per previously reported methods(61). Briefly, the RNA samples were diluted with 1 × TMS buffer to a final concentration of 3-5 nM. Then, droplets of samples (5-10 L) were immediately deposited on APS mica. After 2 min incubation on the specifically modified APS mica surface(41;42), excess samples were washed with DEPC treated water and dried under a flow of Argon gas. AFM images in air were acquired using MultiMode AFM NanoScope IV system (Veeco/Digital Instruments, Santa Barbara, CA) operating in tapping mode.

**Dynamic light scattering**

Apparent hydrodynamic sizes of preassembled triangle, square, and pentagon complexes (10 µM) in 50µL TMS buffer were measured by Zetasizer nano-ZS (Malvern Instrument, LTD) at 25°C. The laser wavelength was 633 nm.

**Flow cytometry Assay**

RAW264.7 cells were detached from the cell culture flask by using a cell scraper. The cells were washed with Opti-MEM medium and aliquot in 1.5 mL Eppendorf tubes at the density of 5 × 10^5^ cells per tube. RNA nanoparticles harboring Cy3-labled CpG DNA or Cy3-labled CpG DNA only were diluted in Opti-MEM medium and incubated with the cells at 37°C for 1.5 h. The cells were vortexed every 30 min during the incubation. After washing with PBS, the cells were resuspended in PBS and the intensity of fluorescence was determined by FACSCalibur flow cytometer (BD Biosciences, San Jose, CA).

**Cytotoxicity assay**

The cytotoxicity of RNA nanoparticles harboring CpG ODNs was evaluated with an MTT assay kit (Promega, Madison, WI), according to the protocol provided by the manufacturer. Briefly, RAW 264.7 cells were seeded at 96 well plates and cultured overnight at 37°C in humidified air containing 5% CO_2_. RNA nanoparticles harboring CpG ODNs and controls were dissolved in fresh cell culture medium at the indicated concentrations and added to the cells for incubation at 37°C for 24 h. Then, 15 µL of the dye solution was added to each well, followed by a 4 h incubation at 37°C, Next, 100µl of the solubilization solution was added to each well and the plate was future incubated at room temperature on a plate shaker until the formazan crystals were completely solubilized. The absorbance was measured at 570 nm using a microplate reader. The cell viability was calculated relative to the absorbance of the cell only control (viability of cell only control = 1).

**Supplementary figure 1**. RNA polygons dissociation constant determination at equilibrium state. These are 7% native PAGE titration data for formation of triangle (a), square (b), and pentagon (c) polygons. Below, the gels is the plot used to determine the equilibrium concentration for each polygon which were then used to calculate the apparent dissociation constant.

**Supplementary figure 2**. Sequences and secondary structures of RNA polygons. RNA polygons and quantified assembly yields for triangle (a), square (b) and pentagon (c).

**Supplementary figure 3**. AFM images of RNA polygons. AFM images of RNA polygons. Population distribution of RNA triangle (a), square (b), and pentagon (c) polygons in 0.5 μm^2^ area of AFM mica surface. Error represents counts from several independent images.

**Supplementary figure 4.** Secondary structure of triangle-CpG nanoparticle and characterization. (a) 2D structure of RNA triangle harboring 3 CpG adjuvants. (b) This is 4% agarose gel showing assembly of RNA triangle nanoscaffold with CpG adjuvants. (c) DLS characterization of the triangle-3CpG complex showing apparent hydrodynamic diameter of around 14 nm. The error represents standard deviation from several independent measurements.

Note: Arrows indicate to base pairs that have been deleted in RNA triangle nanoparticle usedfor animal, confocal microscope, and cytotoxicity studies.

**Supplementary figure 5.** Secondary structure of square-CpG nanoparticle and characterization. (a) 2D structure of RNA square harboring 4 CpG adjuvants. (b) This is 4% agarose gel showing assembly of RNA square nanoscaffold with CpG adjuvants. (c) DLS characterization of the square-4CpG complex showing apparent hydrodynamic diameter of around 15 nm. The error represents standard deviation from several independent measurements.

**Supplementary figure 6**. Secondary structure of pentagon-CpG nanoparticle and characterization. (a) 2D structure of RNA pentagon harboring 5 CpG adjuvants. (b) This is 4% agarose gel showing assembly of RNA pentagon nanoscaffold with CpG adjuvants. (c) DLS characterization of the pentagon-5CpG complex showing apparent hydrodynamic diameter of around 16 nm. The error represents standard deviation from several independent measurements.

**Supplementary figure 7**. Effect of RNA-CpG adjuvants on RAW264.7 cell viability. The cells were incubated with different concentration RNA polygons only, RNA polygons-CpG complexes, and free CpG.

**Supplementary figure 8**. Binding effect of RNA polygons harboring CpG adjuvants to RAW264.7 cells. Concentration dependent binding of (a) triangle-3CpGs, (b) square-4CpGs, and (c) pentagon-5CpGs nanoparticles.

**Supplementary figure 9**. Serum stability assay of RNA polygons coupled with CpGs motifs. Preassembled complexes (1 μM) of RNA triangle, square and pentagon (2’-F modified) harboring DNA CpG were incubated in RPMI-1640 medium containing 10% fetal bovine serum (Sigma). Aliquots (10 μL) were taken at 0 hr, 1 hr, 3 hr, 6 hr, 8hr and 16 hr time points after incubation at 37°C, followed by analysis using 6% native PAGE gel.
